# Florastamin Uptake on PSMA PET/CT as an Imaging Biomarker of Tumor Aggressiveness in Prostate Cancer

**DOI:** 10.3390/diagnostics16162536

**Published:** 2026-08-12

**Authors:** Yeongjoo Lee, Joo Hyun O, Seunggyun Ha, Chansoo Park, Dongho Shin, Hyong Woo Moon, Ji Youl Lee

**Affiliations:** 1 Division of Nuclear Medicine, Department of Radiology, College of Medicine, Seoul St. Mary’s Hospital, The Catholic University of Korea, Seoul 06591, Republic of Korea; soap2222@cmcnu.or.kr (Y.L.); seunggyun.ha@gmail.com (S.H.); 2 Research Institute of Labeling, FutureChem Co., Ltd., Seoul 04794, Republic of Korea; 3 Department of Urology, College of Medicine, Seoul St. Mary’s Hospital, The Catholic University of Korea, Seoul 06591, Republic of Korea

**Keywords:** prostate cancer, florastamin, East Asian, PET/CT

## Abstract

**Background**: This study aimed to evaluate the association between the uptake on F-18 florastamin PET/CT, which targets the prostate-specific membrane antigen (PSMA), and the histopathological grade of the primary tumor in newly diagnosed prostate cancer patients. **Methods**: As a subset analysis of a phase III multicenter trial of florastamin PET/CT in East Asian men, the data from a single institution of pathologically confirmed prostate cancer cases were reviewed. The maximum standardized uptake value (SUVmax) was measured from the primary tumor on florastamin PET/CT images of 110 participants. The International Society of Urological Pathology (ISUP) grade group obtained from surgery or biopsy was classified into three risk groups: low (ISUP 1), intermediate (ISUP 2–3), or high (ISUP 4–5). Correlation was tested between the SUVmax of the prostate cancer and the ISUP-based risk. The PSA level and Prostate Imaging Reporting and Data System (PIRADS) score from MRI were also assessed for association with the SUVmax and ISUP-based risk. **Results**: The SUVmax of the primary tumor and the PSA level showed significant positive correlation with the ISUP-based risk (ρ = 0.541, *p* < 0.001; ρ = 0.328, *p* = 0.001, respectively). The tumor SUVmax increased with higher PIRADS scores (ρ = 0.516, *p* < 0.001). **Conclusions**: The SUVmax of the primary tumor on florastamin PET/CT was associated with the ISUP-based risk in East Asian men with prostate cancer, suggesting that it could be an imaging biomarker of tumor aggressiveness.

## 1. Introduction

Prostate cancer is one of the most commonly diagnosed malignancies among men worldwide. Its incidence and prevalence have been steadily increasing in Asian countries including Korea, coinciding with the population aging and the broader implementation of prostate-specific antigen (PSA)-based screening programs [1,2,3,4].

In contemporary clinical practice, therapeutic options for localized prostate cancer ranges from active surveillance to radical prostatectomy, radiotherapy, and systemic treatment. The tumor grade, typically assessed by the Gleason score and the International Society of Urological Pathology (ISUP) grade group system, are fundamental for prognostication and treatment selection in localized prostate cancer [5,6,7], but these histopathologic classifications are inherently limited by biopsy sampling errors and intra-tumoral heterogeneity. Discrepancies between the biopsy findings and the final radical prostatectomy specimens are common, especially in patients with intermediate-risk disease [8].

Clinical risk assessment tools that incorporate prostate-specific antigen (PSA) levels and multiparametric magnetic resonance imaging (mpMRI) using the Prostate Imaging Reporting and Data System (PIRADS) have significantly enhanced the prediction of local stage and tumor grade [9]. However, they still underestimate or overestimate clinically significant prostate cancer in a substantial number of men and fail to provide a truly quantitative measure of the intraprostatic tumor burden or biological aggressiveness.

In recent years, prostate-specific membrane antigen (PSMA) positron emission tomography/computed tomography (PET/CT) has emerged as the imaging method of choice for staging of high risk patients and identifying tumor recurrence. F-18 labeled tracers including F-18 PSMA-1007, flotufolastat, fluorocholine, and fluciclovine have demonstrated high detection rates for clinically significant intraprostatic lesions. Additionally, these tracers have shown significant associations between the tracer uptake and established markers of disease aggressiveness, including the Gleason score (GS)/ISUP grade, PSA level, and the d’Amico classification [10,11,12,13].

F-18 florastamin is a novel F-18-labeled small-molecule ligand with high affinity for PSMA, enabling PET/CT imaging of PSMA-expressing primary and metastatic prostate cancer lesions [14,15]. The association between florastamin uptake and histopathological parameters such as tumor grade in treatment-naïve primary disease has not been studied. Reports on the primary prostate tumor uptake on PSMA PET/CT and its association with the tumor pathology are very limited in the Asian population. Therefore, this study aimed to evaluate the correlation between florastamin accumulation in primary tumor and the histopathological grade in patients with prostate cancer.

## 2. Materials and Methods

### 2.1. Patients

The included patients were prospectively enrolled Korean adult males with elevated PSA levels who agreed to undergo florastamin PET/CT as a part of a multicenter phase 3 clinical trial (NCT05004285). In this study, we evaluated consecutive patients who visited the urology department of Seoul St. Mary’s Hospital from January 2020 to November 2024. All participants had no prior prostate-related history, and all had pathologic confirmation. For this study, benign conditions such as prostatic intraepithelial neoplasia, chronic prostatitis, or nodular hyperplasia were excluded. Written informed consent was obtained from all patients. The hospital’s Institutional Review Board approved this study as a retrospective subset analysis of the trial cohort (KC26RISI0094, KC21MDDT0167), and the requirement for additional informed consent for this analysis was waived due to the retrospective design.

### 2.2. Florastamin PET/CT Acquisition

Florastamin was prepared as previously described [16]. After administering a mean dose of 379.6 ± 9.3 MBq (10.2 ± 0.3 mCi), images were acquired 90 min later using a combined PET/CT system (Discovery 710D, GE Healthcare, Waukesha, WI, USA). The acquisition time for each bed position was 3 minutes. All patients were positioned supine during the PET/CT scans. Non-enhanced low-dose CT began at the orbitomeatal line and extended to the proximal thigh, following a standard protocol of 120 kVp, variable mAs adjusted by topographic imaging, and a slice thickness of 2.5 mm. PET scans of the same body region were performed immediately after the CT. CT data were utilized for attenuation correction, and images were reconstructed using a standard ordered-subset expectation maximization algorithm. 

### 2.3. MRI Acquisition

Standard prebiopsy MRI examinations were conducted using 3-T MRI systems (Magnetom Verio, Siemens, Erlangen, Germany, and Ingenia, Philips, Best, The Netherlands) equipped with a pelvic-based array coil. Multiparametric MRI included sagittal, coronal, and axial T2-weighted imaging, axial T1-weighted imaging, axial diffusion-weighted imaging, and dynamic contrast-enhanced sequences.

### 2.4. Image Analysis

PET/CT images were reviewed using XD3 (Mirada Medical, Oxford, UK). Regions of interest (ROIs) were manually outlined around the prostate to exclude bladder activity, and the maximum standardized uptake value (SUVmax) of the tumor was from the 90-minute images. The highest SUVmax observed across the prostate was recorded and used for analysis. If activity from the prostatic urethra was suspected in the midline of the prostate, it was excluded from the tumor uptake assessment.

The PIRADS score from the mpMRI images were obtained by an experienced radiologist. The PIRADS score was not available in one patient who declined to participate. If no lesions were found on the MRI, it was recorded as PIRADS group 1; if the MRI was performed after a confirmed diagnosis of prostate cancer, it was classified as PIRADS 5. Cases with multifocal findings for PIRADS 4 and 5 were classified as PIRADS 5.

### 2.5. Histopathology Analysis

All patients underwent MRI, PET/CT, and biopsy within a one-month interval. For patients with visible lesions on either PET/CT or MRI, targeted biopsies were performed by an experienced urologist, in addition to the random 12-core approach on other areas of the prostate gland. Each biopsy sample was labeled separately and reviewed by a board-certified pathologist specializing in urology, particularly prostate pathology, who was blinded to the imaging results. The tumors were classified according to the 2019 ISUP Consensus as follows: grade group 1 (GS ≤ 6), grade group 2 (GS 3 + 4 = 7), grade group 3 (GS 4 + 3 = 7), grade group 4 (GS 4 + 4 = 8, GS 3 + 5 = 8, GS 5 + 3 = 8), and grade group 5 (GS 9–10) [17].

### 2.6. Statistical Analysis

All results are presented as the mean ± standard deviation (SD) and median with a range (minimum–maximum). The International Society of Urological Pathology (ISUP) grade group was used to classify the patients into three ISUP-based risk groups: low risk (grade group 1), intermediate risk (grade groups 2–3), or high risk (grade groups 4–5). Spearman’s rank correlation test was used to evaluate correlations between tumor SUVmax and ISUP grade groups. One-way ANOVA was employed to compare SUVmax among the low-, intermediate-, and high-risk groups. The receiver operating characteristic (ROC) curve was generated to distinguish between clinically significant intermediate/high-risk and low-risk prostate cancer. The optimal SUVmax threshold for distinguishing low-risk from intermediate/high-risk disease was defined as the value that maximized the Youden index (sensitivity + specificity − 1) on the ROC curve. We also analyzed the patients who underwent radical prostatectomy separately. In this subgroup, ISUP grade groups were derived exclusively from the surgical specimen. Statistical analyses and graphs were created using the Statistical Package for Social Sciences (SPSS Statistics 24) and GraphPad Prism 10 (Version 10.6.1). All statistical tests for significance were two-sided, and a *p*-value of less than 0.05 was considered significant.

## 3. Results

This study assessed consecutive florastamin PET/CT performed between January 2020 and November 2024 at a single center, as part of a larger multicenter phase III trial of men suspected of having prostate cancer. Patients whose final pathology revealed prostatic intraepithelial neoplasia, chronic prostatitis, or nodular hyperplasia were excluded from this analysis. A total of 110 patients were included, with histologically confirmed primary prostate cancer, encompassing ISUP grade groups 1–5. All participants underwent florastamin PET/CT and had histologic confirmation by transrectal ultrasound-guided biopsy or robot-assisted radical prostatectomy.

### 3.1. Patient Characteristics

**Figure 1:**
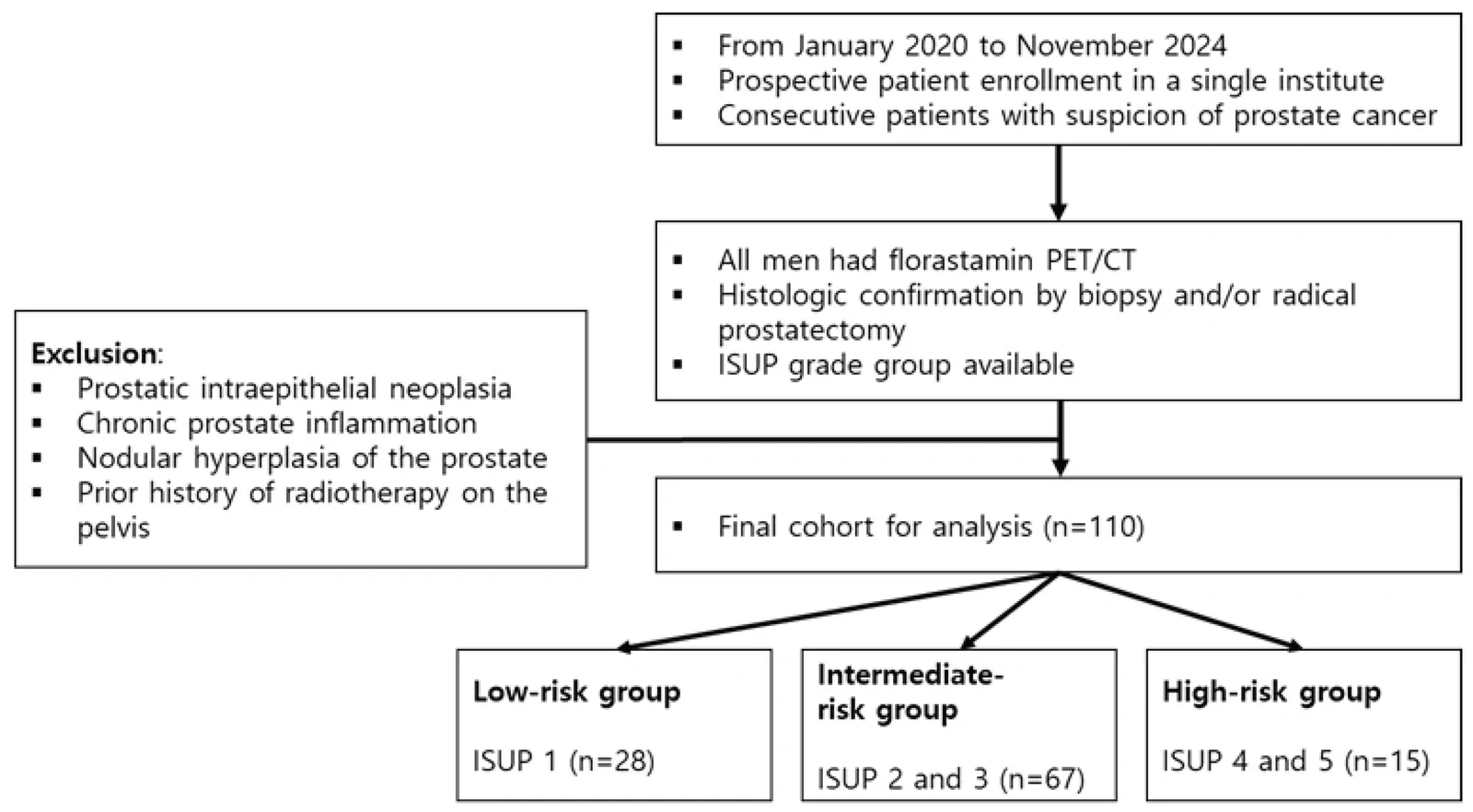
Flowchart of patient recruitment.

Among the participants, 59 underwent robot-assisted radical prostatectomy (RARP), and their postoperative pathology results were referenced. The pathology for the remaining 51 patients was based on the results of the biopsies. For those who underwent surgery, the final pathology was determined from the surgical specimens (Figure 1). The mean patient age was 67.5 ± 8.6 years (range: 40–85), and the mean PSA level was 70.7 ± 45.7 ng/mL. Patients were stratified according to the ISUP-based risk groups: low-risk (*n* = 28), intermediate-risk (*n* = 67), and high-risk (*n* = 15) groups. Overall, 51 patients underwent biopsy alone, while 59 underwent both biopsy and radical prostatectomy. In 7 of the 59 patients (11.8%), the Gleason score from the prostatectomy specimen was higher than that from the biopsy results (Table 1).

### 3.2. Correlation of Florastamin Uptake with ISUP-Based Risk and PSA Level

**Figure 2:**
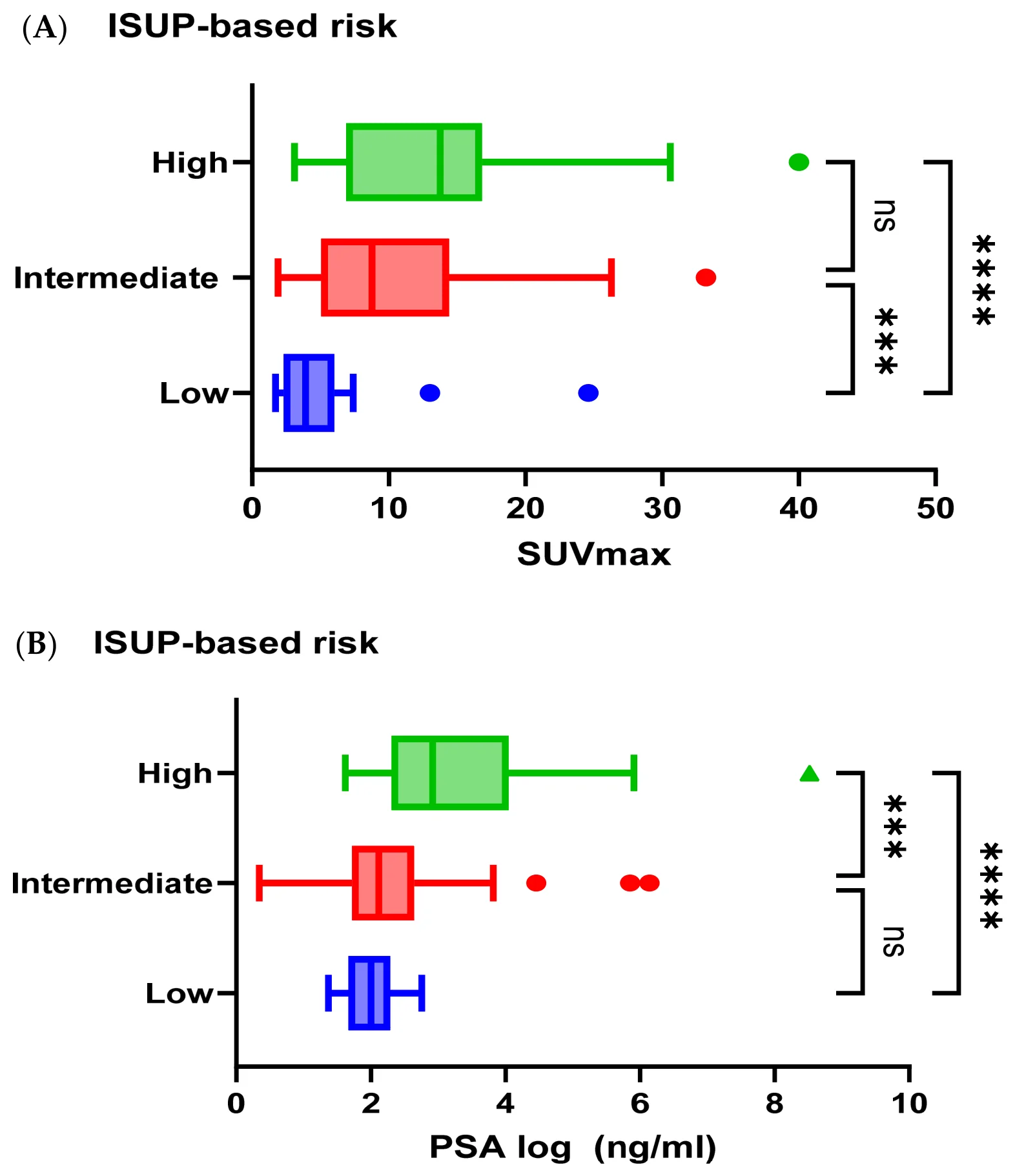
Tumor SUVmax and PSA values (log scale) of the ISUP-based risk groups. (**A**) Box-and-whisker plots demonstrate positive correlation between ISUP-based risk and tumor SUVmax (ρ = 0.541, *p* < 0.001). (**B**) A significant positive correlation is found between the PSA level and the ISUP-based risk group (ρ = 0.328, *p* = 0.001). Low risk (ISUP grade group 1); intermediate risk (ISUP grade groups 2–3); high risk (ISUP grade groups 4–5). Statistical significance annotations are shown as follows: ns, not significant (*p* ≥ 0.05); *** *p* < 0.001; **** *p* < 0.0001.

In the final cohort of 110 patients, tumor SUVmax exhibited a stepwise increase across the pathological risk strata and also the PIRADS scores (Table 2 and Table 3). The ISUP-based low-risk group displayed the lowest PSMA uptake intensity on PET/CT, with a mean SUVmax of 4.3 ± 2.8. The intermediate-risk group had a higher mean SUVmax of 10.3 ± 9.5 (median, 7.5), while the high-risk group showed the highest mean SUVmax of 12.8 ± 7.3 (median, 13.7). The ISUP-based risk and tumor SUVmax had a correlation coefficient of 0.541 (*p* < 0.0001), indicating a statistically significant higher tracer uptakes in higher histologic grades. A significant positive correlation was found between the PSA level and the ISUP-based risk as well (ρ = 0.328, *p* = 0.001) (Figure 2). There was also a positive correlation between the PSA level and the SUVmax (ρ = 0.379, *p* < 0.001). In the high-risk group, six of fifteen patients demonstrated relatively low intraprostatic florastamin uptake with SUVmax < 10 (average SUVmax: 6.15, range: 3.1~8.3), and the intensity was even lower than that of the liver in two patients. Among the six patients with relatively low intraprostatic PSMA uptake, one had lymph node metastasis and two had multiple bone metastases.

In a subgroup of 59 patients who underwent radical prostatectomy, SUVmax of the primary tumor showed similar findings with significantly higher values in intermediate- and high-risk groups compared with the low-risk group (one-way ANOVA, *p* = 0.022) (Supplementary Figure S1).

**Figure 3:**
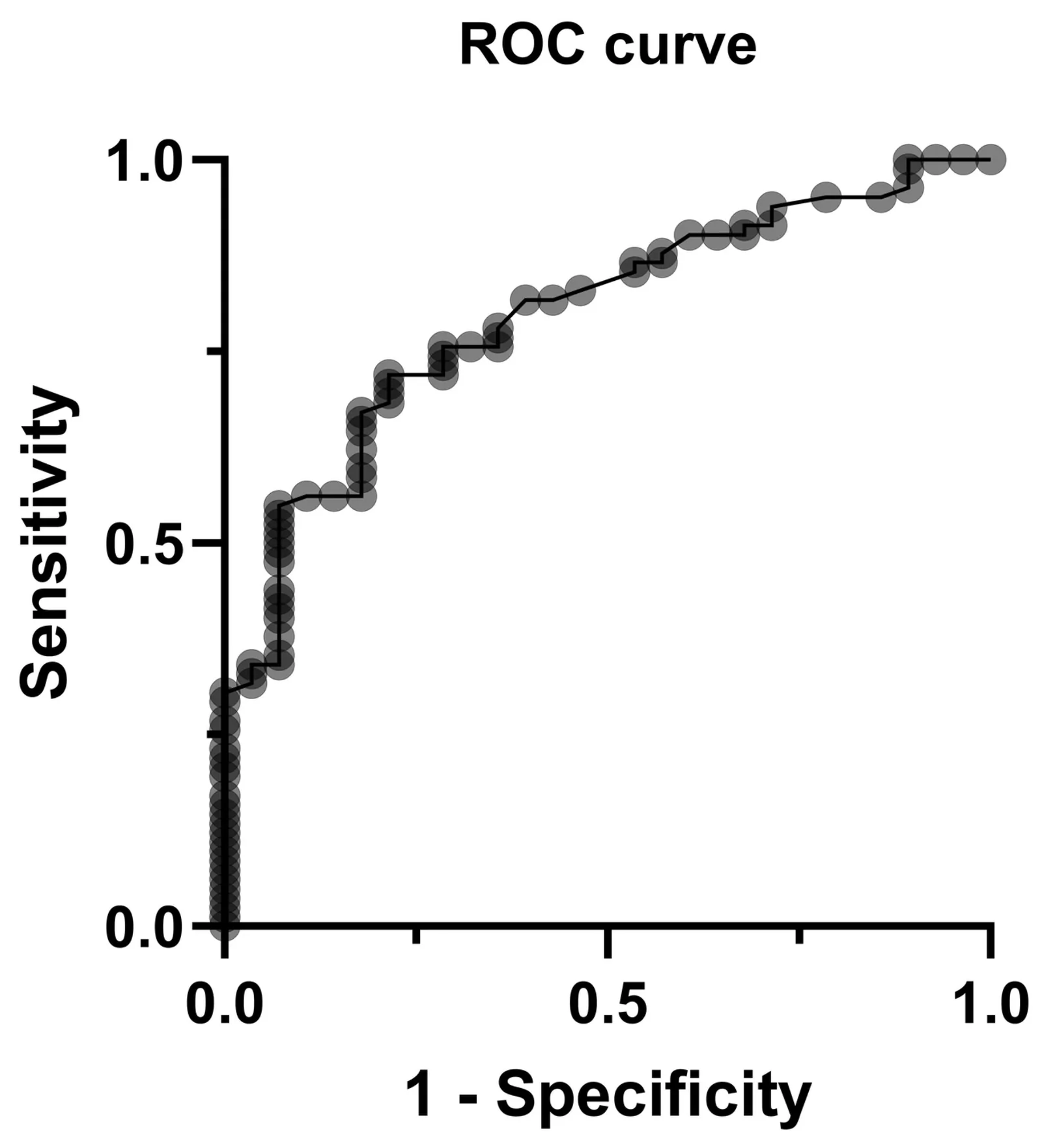
Receiver operating characteristic (ROC) curve of tumor SUVmax for discriminating intermediate- and high-risk groups from the low-risk group. The area under the ROC curve (AUC) was 0.797 (standard error, 0.045; 95% confidence interval, 0.709–0.885; *p* < 0.0001).

ROC analysis was performed to distinguish the intermediate- and high-risk groups (*n* = 82) from the low-risk group (*n* = 28). The area under the ROC curve (AUC) was 0.797 (standard error, 0.045; 95% confidence interval, 0.709–0.885; *p* < 0.0001), indicating good discriminatory performance of tumor SUVmax for identifying clinically significant prostate cancer (Figure 3). The optimal SUVmax cut-off determined by the Youden index was 11.15, providing a sensitivity of 0.587 and a specificity of 0.875.

### 3.3. Correlation of Tumor SUVmax with PIRADS Categories

**Figure 4:**
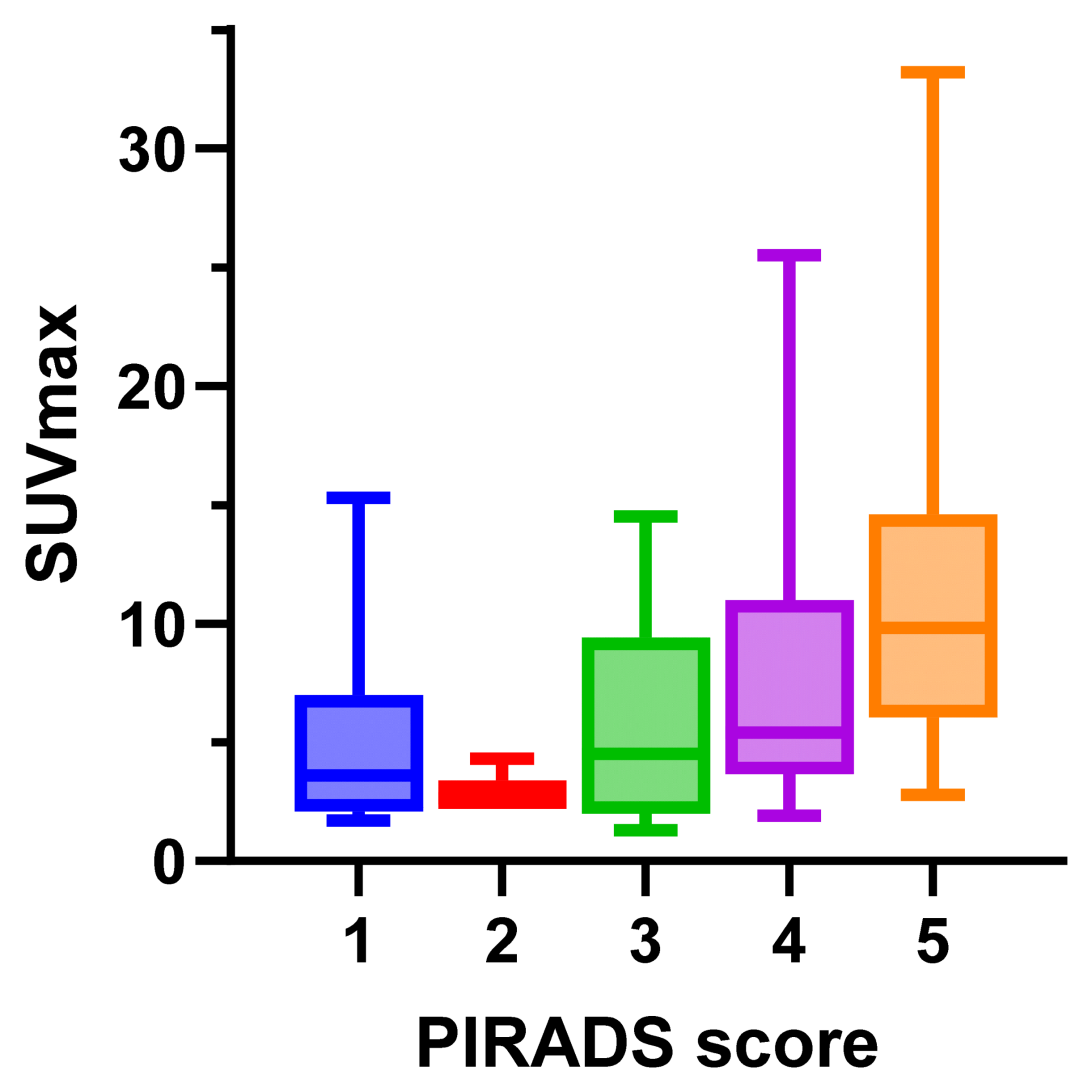
Tumor SUVmax according to PIRADS score. Box-and-whisker plots show an overall increase in tumor SUVmax corresponding to higher PIRADS scores (ρ = 0.516, *p* < 0.001). The median SUVmax values for PIRADS 1 to 5 are 3.6, 2.7, 4.5, 5.4, and 9.8, respectively.

**Figure 5:**
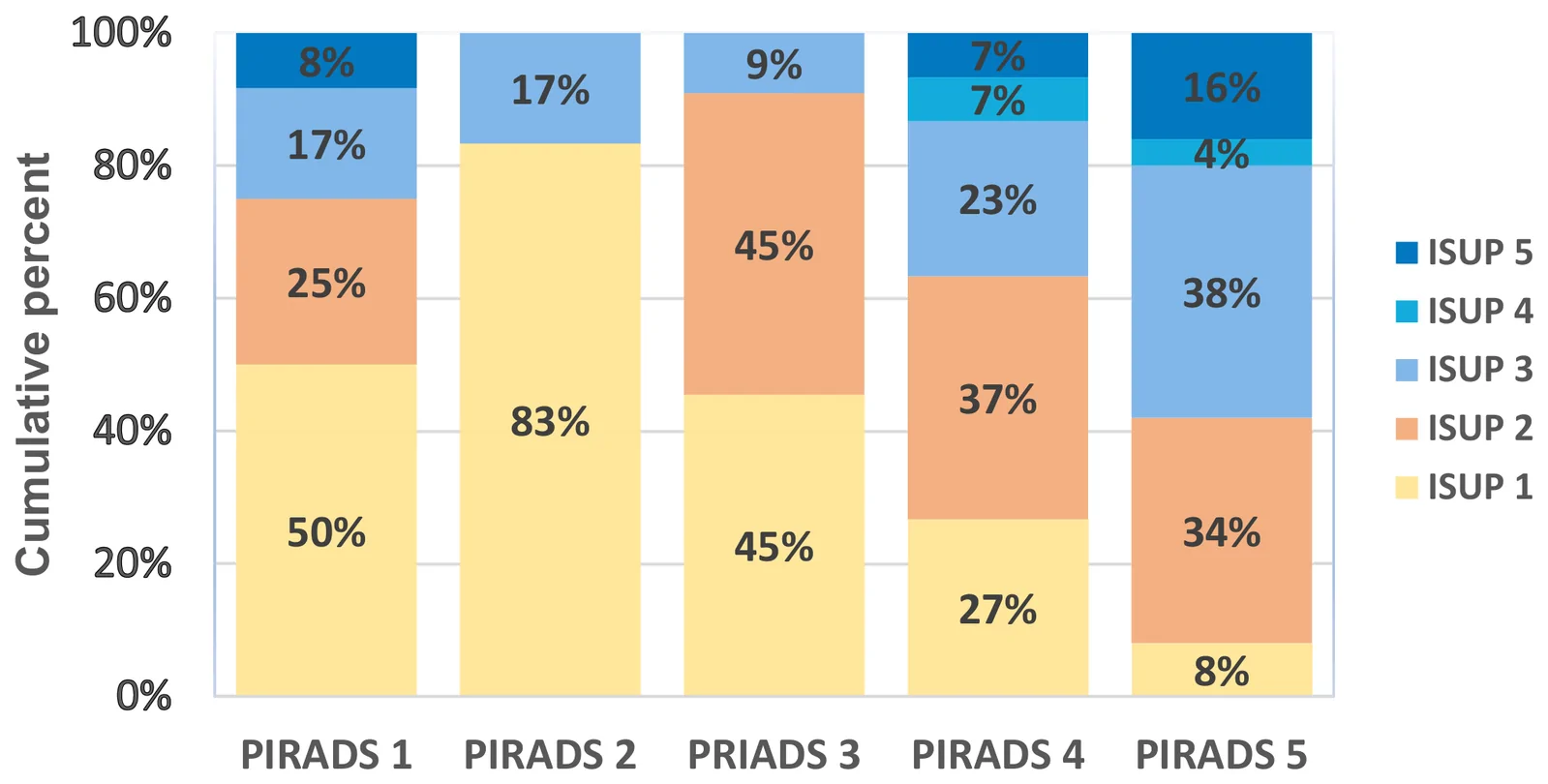
A 100% stacked bar chart illustrating the distribution of ISUP grade groups (1–5) by the PIRADS scores. Higher PIRADS categories exhibited a greater proportion of higher ISUP grade groups. Notably, even within PIRADS 1, 25% of the individuals had ISUP grade group 4 or 5 lesions. Similarly, 17% of the patients with PIRADS 2 were identified to have ISUP grade group 4 lesions.

SUVmax also increased progressively with higher PIRADS scores in the overall cohort (ρ = 0.516, *p* < 0.001). The florastamin uptake measurements were as follows: PIRADS score of 1, mean SUVmax of 4.8 ± 1.1, and a median of 3.6; PIRADS score of 2, mean SUVmax of 2.8 ± 0.3; PIRADS score of 3, mean SUVmax of 5.5 ± 1.4; PIRADS score of 4, mean SUVmax of 7.8 ± 1.1; PIRADS score of 5, mean SUVmax of 12.3 ± 1.5 (Figure 4). Notably, in 96.4% (27/28) of patients with an SUVmax exceeding 12, corresponding to a PRIMARY score of 5, the pathology results indicate clinically significant prostate cancer (Gleason score of 7–9), regardless of the PIRADS score. Higher PIRADS categories correlated with a greater proportion of higher-ISUP grade groups (Supplementary Table S1). Remarkably, even with a PIRADS score of 1, 25% of the individuals had prostate cancer belonging in ISUP grade groups 4 and 5. Similarly, lesions with a PIRADS score of 2, 17% of the lesions were later identified to have ISUP grade group 4 (Figure 5).

**Figure 6:**
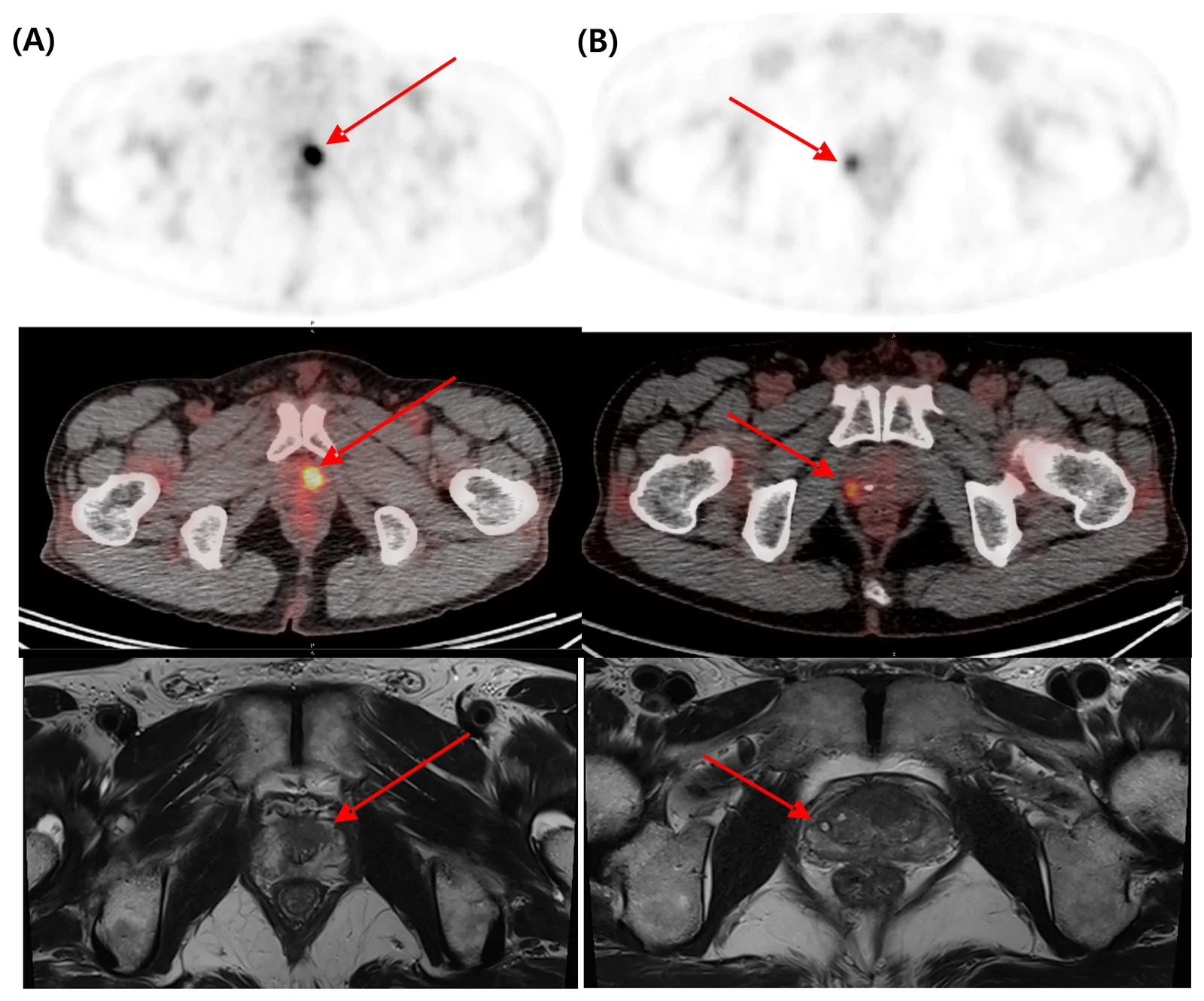
Two examples illustrate that increased florastamin uptake is associated with the higher-risk group. (**A**) A 73-year-old man with a PSA level of 45.3 ng/mL and an SUVmax of 30.6 presented with a 1.5 cm PIRADS 5 lesion in the left transition zone at the apical level (red arrow). He underwent radical prostatectomy, and final pathology confirmed a high-risk lesion (Gleason score of 8 (4 + 4), ISUP grade group 4). (**B**) A 72-year-old man with a PSA level of 7.07 ng/mL and an SUVmax of 3.8 had an approximately 1.0 cm T2 low-signal mass in the right peripheral zone of the prostate (red arrow), classified as PIRADS 5 (cT2N0). He also underwent radical prostatectomy, and final pathology showed a low-risk lesion (Gleason score of 6 (3 + 3), ISUP grade group 1).

When applying a combined threshold of SUVmax ≥ 10 and PSA ≥ 10 ng/mL on florastamin PET/CT to detect clinically significant prostate cancer, the specificity was 96.4% and the positive predictive value was 95.7%. Cases demonstrating the association between florastamin uptake and ISUP-based risk group are presented in Figure 6.

## 4. Discussion

Our study demonstrates a statistically significant overall positive correlation between intraprostatic florastamin uptake and histopathological grade in primary prostate cancer, with higher SUVmax values observed in tumors with more advanced ISUP-based risk groups. These findings align with previous reports on other PSMA-targeted PET tracers, such as F-18 PSMA-1007, which have shown that increased intraprostatic PSMA uptake is associated with higher Gleason scores/ISUP grades, elevated PSA levels, and greater clinical risk categories [10,12,13]. The present data extend this concept to florastamin PET/CT by showing that its uptake correlates with ISUP-based risk in treatment-naïve primary tumors in East Asian men, who frequently present with advanced stage and are known to have with distinct genomic profiles [18].

Currently, ISUP grade group 1 is generally managed as a low-risk disease, most often with active surveillance, and curative treatment is reserved for selected patients. Patients with ISUP grade group 2–3 tumors usually receive definitive local therapy, or are sometimes managed with short-term androgen deprivation therapy (ADT), while patients with ISUP grade group 4–5 tumors are considered to be in high- or very-high-risk groups with aggressive multimodal approaches, including extended surgery or dose-escalated radiotherapy plus long-term ADT [19]. Accurately identifying high-risk prostate tumors is crucial at the time of initial diagnosis, as treatment options depend on the tumor’s aggressiveness.

Previous studies have demonstrated the usefulness of PSMA PET-targeted biopsy [20,21]. Prostate MRI PIRADS scores generally aligned with ISUP grading; however, a few cases with low PIRADS scores displayed aggressive tumor features, highlighting the discordance between the image findings and histological grades. Additionally, discrepancies between the preoperative biopsy and postoperative surgical pathology reports, “grade shifting”, were noted in three cases, underscoring the limitations of biopsy procedures. One patient with a PI-RADS score of 1 and two patients with a PI-RADS score of 3 had SUVmax values above 12 and were subsequently diagnosed with prostate cancer. Therefore, the uptake of florastamin and the PIRADS score should be interpreted as complementary markers for accurately evaluating preoperative biopsy sites and overall disease status.

Uprimny et al. demonstrated in their study that the intensity of tumor-related tracer uptake on Ga-68 PSMA PET/CT correlates with the PSA concentration and Gleason score in newly diagnosed prostate cancer [22]. When Gleason scores (GSs) were considered alongside tumor-related tracer uptakes, a similar incremental trend in SUVmax was observed; higher values corresponded to increased GS/ISUP grades, a pattern noted in our cohort. Ulas et al. investigated the combined use of PSMA-PET imaging and conventional staging criteria, suggesting that integrating PSMA-derived imaging parameters into established clinical models may enhance prognostic accuracy in prostate cancer [23]. These reports suggest the value of PSMA-PET in risk stratification and align with the findings of the present study, which specifically focused on SUVmax as the primary quantitative measure in PET.

PSMA PET/CT demonstrates high sensitivity for detecting prostate cancer; however, several studies have indicated that it may not identify a subset of high-grade tumors, particularly those with Gleason scores of 9–10 [24]. Tumor heterogeneity can result in variable PSMA expression, with some biologically aggressive cancers showing low or absent PSMA uptake. There is also a previous report that East Asian tumors show lower rates of ERG fusions and TP53/PTEN mutations, and higher frequencies of FOXA1 and SPOP alterations compared with Western or South Asian populations [25]. These genomic alterations in the East Asian populations, which may affect androgen receptor signaling, could also influence PSMA expression on PET imaging. Poorly differentiated tumors may exhibit altered PSMA expression due to changes in tumor biology or the surrounding microenvironment. A shift toward a neuroendocrine phenotype, which is known to be accompanied by concurrent TP53 and RB1 loss, is associated with markedly decreased or heterogeneous PSMA expression [26]. Additionally, variations in tumor vascularization, cellular density, or predominant metabolic pathways may influence PSMA uptake, further contributing to reduced tracer accumulation in some high-grade lesions.

Our cohort included six patients (5.4%) with high-risk tumors with discordantly low florastamin uptake but markedly elevated PSA levels and/or metastatic disease, confirming that tumor aggressiveness does not always correspond to high PSMA expression. These observations suggest that, in PSMA-low or discordant cases, serum PSA may provide complementary information for identifying biologically aggressive prostate cancer and vice versa. Additionally, incorporating fluorodeoxyglucose (FDG) PET/CT may help characterize PSMA-negative but FDG-positive diseases, which could represent a distinct and clinically significant disease entity [27,28].

Our study has several limitations. First, it was a retrospective analysis conducted at a single tertiary center. Second, we measured the SUVmax as a single voxel parameter, which may not fully capture intra-tumoral heterogeneity or the total tumor burden. While volumetric PSMA metrics, such as PSMA-positive tumor volume or total lesion PSMA uptake, could offer additional insights [29], they were not evaluated in our study as no method for measuring the volume has been clearly established. Interobserver variability in PSMA PET/CT interpretation and measurements remains unresolved [30,31], and we chose SUVmax for its low variability. We are working on reducing reader variability through internal training sessions and plan on incorporating volumetric PSMA PET metrics in future studies. Third, our SUV measurements are subject to partial volume effects, particularly in small or ill-defined lesions, which may lead to the underestimation of true tracer uptake. We did not conduct a segmental or level-by-level analysis of the tracer-avid regions or the pathology specimens. Instead, we focused on a single SUVmax value across the entire prostate; this approach is not representative of the overall metabolic activity of the tumor. Although the mean SUV could provide a more comprehensive assessment of tumor burden and intra-tumoral heterogeneity, it could also be more susceptible to miscalculation from prostate urethral activity. Consequently, we prioritized a more practical and less variable metric.

Despite these limitations, our study found that florastamin uptake is associated with ISUP-based risk in East Asian men, and demonstrates its potential as an imaging biomarker of PSMA expression. Future multicenter studies with larger cohorts incorporating advanced quantitative PET metrics are necessary to validate these findings and determine whether florastamin-based PET parameters can enhance existing risk models or guide biopsy targeting for refined selection between active surveillance and definitive treatment in patients with prostate cancer.

## 5. Conclusions

Our study demonstrates that the uptake values of the prostate tumor on florastamin PET/CT are associated with the ISUP-based risk in newly diagnosed East Asian men with prostate cancer and suggest SUVmax could be an imaging biomarker of tumor aggressiveness.

## Figures and Tables

**Table 1:** Patient characteristics (*n* = 110).

Characteristics	Values
Age (years)	
Average ± SD	67.5 ± 8.6
Range	40~85
PSA (ng/mL)	
Average ± SD	70.7 ± 45.7
Median	8.6
Range	0.7~5000.0
ISUP grade group	
1	28 (25.5%)
2	36 (32.7%)
3	31 (28.2%)
4	4 (3.6%)
5	11 (10.0%)
Clinical N stage/M stage	
N0/M0	98 (89.1%)
N1/M0	3 (2.7%)
N0/M1	2 (1.8%)
N1/M1	7 (6.4%)
PIRADS	
1	12 (10.9%)
2	6 (5.5%)
3	11 (10.0%)
4	30 (27.3%)
5	50 (45.4%)
Refused MRI	1 (0.9%)

**Table 2:** Tumor SUVmax values according to ISUP-based risk.

ISUP-Based Risk	Patients (*n* = 110)	SUVmax	
		Average ± SD	Median (Range)
Low (ISUP 1)	28 (25.5%)	4.3 ± 2.8	3.8 (3.2–5.4)
Intermediate (ISUP 2/3)	67 (60.9%)	10.3 ± 9.5	7.5 (7.9–12.6)
High (ISUP 4/5)	15 (13.6%)	12.8 ± 7.3	13.7 (8.8–16.8)

**Table 3:** Tumor SUVmax values according to PIRADS.

PIRADS	Patients (*n* = 110)	SUVmax	
		Average ± SD	Median (Range)
1	12 (10.9%)	4.8 ± 1.1	3.6 (1.7–15.3)
2	6 (5.5%)	2.8 ± 0.3	2.6 (2.2–4.3)
3	11 (10.0%)	5.5 ± 1.4	4.5 (1.3–14.5)
4	30 (27.3%)	7.8 ± 1.1	5.4 (1.9–25.5)
5	50 (45.4%)	12.3 ± 1.5	10.4 (2.8–65.6)
N/A	1 (0.9%)	(-)	(-)

## Data Availability

The data from this study are available from the corresponding author upon reasonable request due to privacy and ethical restrictions.

## Supplementary Materials

The following supporting information can be downloaded at https://www.mdpi.com/article/10.3390/diagnostics16162536/s1, Table S1: ISUP grades according to each PIRADS score. Figure S1: Tumor SUVmax according to ISUP-based risk groups in the radical prostatectomy subgroup (*n* = 59). Box-and-whisker plots display SUVmax values according to pathological risk groups derived from radical prostatectomy specimens. A one-way ANOVA showed a significant difference in SUVmax among the risk groups (*p* = 0.0222).

## Abbreviations

The following abbreviations are used in this manuscript:

PET/CT	positron emission tomography/computed tomography
PSA	prostate-specific antigen
ISUP	International Society of Urological Pathology
MRI	magnetic resonance imaging
PIRADS	Prostate Imaging Reporting and Data system
