# Supplementary material for: Florastamin Uptake on PSMA PET/CT as an Imaging Biomarker of Tumor Aggressiveness in Prostate Cancer
Source: Diagnostics (Basel). 2026 Aug 12;16(16):2536. doi: 10.3390/diagnostics16162536 (PMC13511251; doi:10.3390/diagnostics16162536)
Supplement: Supplementary file 1 [file diagnostics-16-02536-s001.zip › diagnostics-4370070-supplementary.pdf]

Supplementary Table S1. ISUP grades according to each PIRADS scores

	PIRADS 1	PIRADS 2	PIRADS 3	PIRADS 4	PIRADS 5
ISUP 1	6	5	5	8	4
ISUP 2	3	0	5	11	17
ISUP 3	2	1	1	7	19
ISUP 4	0	0	0	2	2
ISUP 5	1	0	0	2	8

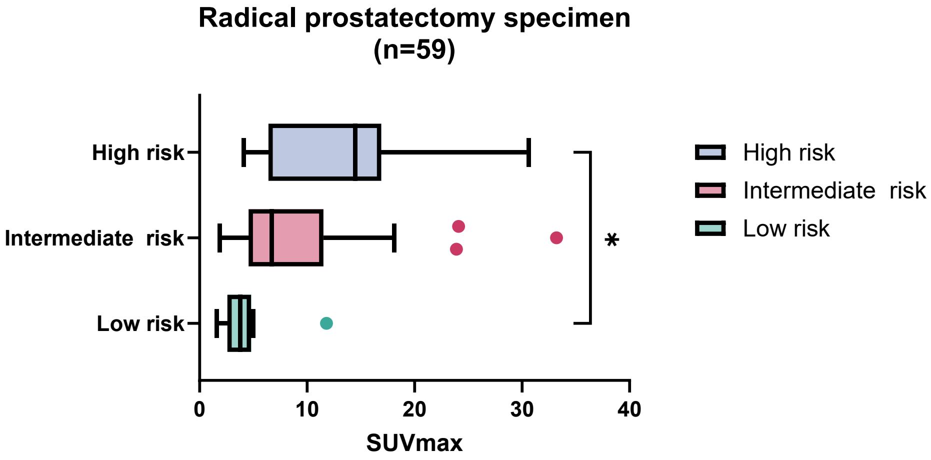

Supplementary Figure S1. Tumor SUVmax according to ISUP-based risk groups in the radical prostatectomy subgroup (n = 59). Box-and-whisker plots display SUVmax values according to pathological risk groups derived from radical prostatectomy specimens. A one-way ANOVA showed a significant difference in SUVmax among the risk groups (p = 0.0222).
